# Storing and Using Health Data in a Virtual Private Cloud

**DOI:** 10.2196/jmir.2076

**Published:** 2013-03-13

**Authors:** Nathan Regola, Nitesh V Chawla

**Affiliations:** ^1^Interdisciplinary Center for Network Science and ApplicationsDepartment of Computer Science and EngineeringUniversity of Notre DameNotre Dame, INUnited States

**Keywords:** medical informatics, HIPAA

## Abstract

Electronic health records are being adopted at a rapid rate due to increased funding from the US federal government. Health data provide the opportunity to identify possible improvements in health care delivery by applying data mining and statistical methods to the data and will also enable a wide variety of new applications that will be meaningful to patients and medical professionals. Researchers are often granted access to health care data to assist in the data mining process, but HIPAA regulations mandate comprehensive safeguards to protect the data. Often universities (and presumably other research organizations) have an enterprise information technology infrastructure and a research infrastructure. Unfortunately, both of these infrastructures are generally not appropriate for sensitive research data such as HIPAA, as they require special accommodations on the part of the enterprise information technology (or increased security on the part of the research computing environment). Cloud computing, which is a concept that allows organizations to build complex infrastructures on leased resources, is rapidly evolving to the point that it is possible to build sophisticated network architectures with advanced security capabilities. We present a prototype infrastructure in Amazon’s Virtual Private Cloud to allow researchers and practitioners to utilize the data in a HIPAA-compliant environment.

## Introduction

### Intended Audience

This paper presents an overview of current challenges in the research community as health care data are utilized to explore personalized medicine and other information technology–related advancements. This work attempts to address an appropriate network and systems architecture to support compliance with Health Insurance Portability and Accountability Act (HIPAA) regulations in the United States in a research environment. The intended audience of the article is computer scientists, network administrators, principal investigators, and managers of research programs that utilize, or want to utilize, protected health information (PHI) in their research programs. The reader is expected to be familiar with basic computer science principles and terms, such as subnetwork, firewall, router, virtual local area network (VLAN), access control lists, and event logging. Additionally, the reader should have a basic understanding of computer system configuration and network architectures. Where appropriate, the paper references material that may be helpful for readers to acquire indepth knowledge of the concepts, but we attempt to explain the necessary knowledge to understand the paper.

### Background

The growing adoption of electronic health records provides a rich source for data mining in order to identify patterns and trends in health care data. Many communities across the United States have or are forming health information exchanges (HIE). Health information exchanges serve as a common hub for data exchange. These organizations enable health care organizations to transfer data by contacting a common hub, the HIE, instead of maintaining connections with numerous peer organizations. Health information exchanges can also provide data to researchers. We expect that this trend will grow since universities (and other research organizations) can reach a legal agreement with the HIE for data exchange instead of negotiating with numerous organizations. The fundamental challenge in enabling such data partnerships is to provide a secure environment for data sharing and adhering to HIPAA, as necessary. A rarely discussed but relevant aspect of creating “a secure environment” in the research community is the ability to define the network and systems architecture to suit the relevant requirements. Organizations, such as hospitals and large medical research complexes, likely have architected their entire information technology infrastructure to support protected health information.

However, in general research environments or enterprise environments, the information technology systems are designed to support several stakeholder groups and their reconfiguration is not undertaken lightly (in our experience). A research university often has at least two types of network security zones for workstations and other computing clients: a zone to support financial systems, student information systems, and general business functions and a zone to support research systems that is generally less restrictive in use (and occasionally open to external parties). Often workstations are placed into one zone. Servers and other centralized services are typically placed in a series of private zones within a data center network, with appropriate access to the other zones to permit, for example, employees to access the financial databases and for students and faculty to access files stored on central servers.

A server to support sensitive health data does not fit into this model, as it is neither an enterprise computing system, such as a financial database, nor a research system that should be accessed by internal and external parties. A system to support the data mining of health information should be a private server that is accessible to only a few authorized individuals, with appropriate support to implement auditing and other requirements for HIPAA compliance. Frequently, provisioning new servers in a data center is a slow process due to the necessity to configure new networks and coordinate the physical installation of hardware. Amazon’s Virtual Private Cloud (VPC) service offering allows researchers to rapidly provision networks and servers. The features of the Amazon VPC cloud service allow network architects to build sophisticated networks, just as they would in a physical environment. Ultimately, universities and other organizations are likely to set up private clouds that allow researchers to rapidly provision networks and servers that suit a variety of needs. However, the concepts and commentary are largely applicable regardless of whether the researchers utilize a private cloud or a public offering such as Amazon.

*Even if the data are anonymous/deidentified*, it is important to implement the relevant privacy, access, and security safeguards as a matter of information security best practices. In this paper, we present our experience in setting up Amazon’s “Virtual Private Cloud offering” [1], a part of Amazon EC2, for health data exchange. We provided a server with Mirth Connect [2], an open source tool that can connect to health information systems (see Appendix 1). We used Mirth Connect to do a pilot run with a health exchange that provided us anonymized and scrubbed data about patients using the HL7 message format [3,4] over an SSL connection (see Appendix 2). We were able to use Mirth Connect to integrate real-time health data. Obviously, security and privacy were of paramount concern, and we wanted to explore the feasibility of creating a HIPAA compliant environment so that other institutions, researchers, and practitioners could potentially work with protected health information (PHI). Our goal with this paper is to demonstrate the necessary steps to set up a Mirth Connect server (although other health data exchange tools could be used) within the Amazon VPC environment, addressing HIPAA compliance where necessary. We should note that three groups of students from the Healthcare Analytics course at the University of Notre Dame successfully incorporated the Mirth Connect framework into their group projects. The students successfully completed the relevant Human Subjects training as part of the curriculum as well. While the health exchange provided simulated data to the students, we believe the infrastructure allows for anonymized patient data.

### HIPAA Requirements

Broadly speaking, HIPAA (1996) requires the US Department of Health and Human Services to adopt national standards for health care transactions and code sets, unique health identifiers, privacy, and security [5]. The transaction and code set standards discuss the content and format for various types of transactions including claim filings, claim status, payment advice, and other types of electronic data transactions that occur between specific types of health providers, health plans, and data clearinghouses. The rules that govern transactions and code sets are maintained by the Centers for Medicare & Medicaid Services [6]. These rules primarily concern software vendors, data clearinghouses, and health plans and apply regardless of the computational environment. This paper assumes that the organization is formatting transactional data according to the standards for data exchange, as these transactions are created within applications and their implementation is independent of the server and network architecture. This work does not discuss the various data exchange formats since the authors are not developing data exchange software or proposing alternative data exchange standards. This work focuses on the privacy and security rule, as their implementation depends on the computational environment that is utilized to host the servers and network. The privacy rule and security rule mandate standards for the protection of data and accompanying monitoring and auditing to ensure that the protections are functioning adequately. Compliance with the privacy rule and security rule became mandatory in 2003 and 2005, respectively, with a 1-year extension for small health plans.

### Privacy Rule Overview

The intent of the Privacy Rule was to ensure that patients’ privacy was respected as electronic transactions increasingly shared data across many organizations. The Privacy Rule [7] component of HIPAA broadly concerns the use of individually identifiable health information, which is frequently referred to as “protected health information” (PHI). The law is designed to ensure that consumers’ PHI is handled appropriately within the health organization and only shared with outside entities according to the uses permitted by law. The Privacy Rule largely focuses on organizational and legal issues, such as requirements for disclosure accounting, permissible PHI disclosures, and other issues surrounding the privacy and disclosure of PHI. In a research environment, this paper assumes that the organization is entitled to the data and does not need to exchange the data with other entities. This paper does not focus on the organizational and legal requirements surrounding data disclosure to third parties. It is worth noting that the Privacy Rule and Security Rule mandate that an organization appoint a privacy officer and a security officer. Additionally, the organization is required to maintain policies that are consistent with HIPAA (and any state and local laws). The Privacy Rule and Security Rule requirements are applicable regardless of whether an organization maintains their own infrastructure or utilizes cloud computing. However, this paper discusses the more interesting information technology aspects of implementing HIPAA compliance in a cloud computing environment—the challenges of addressing the safeguards in the Security Rule.

### Security Rule Overview

The security of the server depends on the physical security of the server and network, the operators and users of the server, and the configuration and management of the applications, operating system, and network. Examining this from a HIPAA compliance standpoint, there are several sections in the Security Rule that we address, including “Risk Analysis and Management”, “Administrative Safeguards”, “Physical Safeguards”, and “Technical Safeguards”.

#### Use Cases

Several use cases serve to highlight the obstacles to utilizing enterprise and research computing environments.

##### Research Health Data

Assume that a researcher from organization O_1_ wants to obtain PHI from organization O_2_ for a research project and wants to collaborate on the project with collaborator C_1_ and collaborator C_2_ at organization O_2_ and O_3_, respectively. Also assume that the requisite permission for sharing the PHI is available. It is determined by the Information Security department at the researcher’s organization that the PHI should be stored in encrypted files on a server with server access granted to the researcher and collaborator C_1_ and C_2_. Personnel at O_1_ could create accounts for O_2_ and O_3_, but all remote users (through a virtual private network V_1_) at O_1_ have network access to all systems at O_1_ on the default network N_1_. The ideal solution would be to create a new network N_2_ and a new remote user group, V_2_ and allow only users in V_2_, a separate VPN (virtual private network) group, to access N_2_. While these changes would be possible at many organizations, they would require the cooperation of personnel in various departments and manual changes, since the ability to create this architecture is not exposed to the researcher. Utilizing a cloud solution, the researcher could set up a network N_2_ at Amazon and an accompanying VPN V_2_ for the 3 users—the researcher, C_1_, and C_2_. This setup should be provisioned rapidly and easily deleted when the project was complete.

##### Health Data Class Projects

Assume that a researcher wants to set up a system to enable a class of 20 students to work in teams of 4 in miniature data mining projects, utilizing electronic health care records (EHR), albeit deidentified. Each group is allowed access to some element of EHR and not to all, and no group is allowed access to the entire EHR. To ensure that groups do not attempt to access other groups’ data and code and to ensure that “the principle of least privilege” is implemented, five separate networks, named N_1_ through N_5_ will be created, and five separate VPNs will be created, named V_1_ through V_5_. Network N_1_ will be accessible only via VPN V_1_, and network N_2_ will be accessible only via VPN V_2_, and so on, since users assigned to network N_2_ do not need to access N_1_. At the conclusion of the competition, the networks and VPNs will be deleted. Purchasing a physical server for one semester is an expensive proposition (assuming that five virtual machines are utilized so that only one physical server is required). Requesting the provisioning of five networks and five VPN groups, along with the associated configuration, for a semester is likely to be considered a large request, since the system is not a permanent enterprise system. Utilizing cloud computing, this task can be provisioned rapidly and even scripted for repeated use (ie, for the next time the course is offered). The virtual machines are leased for the exact time that they are needed, while the onsite solution requires the upfront purchase of a physical server.

### Why Use the Cloud?

While it is possible to build similar architectures in private data centers, the lead time required to support “research” activity in an enterprise data center can be significant, since the enterprise’s resources and policies are aimed toward supporting enterprise applications, such as accounting, human resources, etc. Often, research computing environments, such as campus clusters, are configured in a lower security setting (since they are optimized for maximum performance and rapid troubleshooting) and do not support the physical security standards necessary for HIPAA since various individuals may be permitted to access data centers and servers. Research computing data center space is typically optimized for rapid troubleshooting and high performance, with many physically unlocked racks accessible by various individuals. Strong physical security standards would likely dictate that sensitive systems are placed in locked racks, separate from the main data center space, so that physical access to the sensitive systems can be audited. Additionally, research computing environments typically do not allow researchers to provision private networks, configured to their needs, within a few days. From a technical perspective, research computing centers could, and probably will, provide a middleware software solution that will eventually enable researchers to create their own customized networks and virtual servers (a private cloud). However, this type of solution is not currently available at many institutions. After private clouds are available at research institutions, the discussion raised in this work should aid researchers in determining if their organization’s solution is appropriate for hosting data regulated by HIPAA.

If a research computing center does not have a private cloud, researchers are forced to acquire physical servers, assuming that appropriate physical security can be arranged. However, the time required to obtain physical servers and coordinate their installation can range from a few days to a month or more (including the time necessary to arrange meetings and plan connectivity). In this situation, researchers are left with the choice of requesting space in the enterprise data center or attempting to increase the security posture of the research computing environment. The authors have typically had to pursue the former option, as increasing the security posture of a research computing environment is a significant investment of resources and would serve only a few users.

Enterprise data centers at research institutions often have many internal networks in order to partition the enterprise wide area network into many private networks for specific purposes. For example, payment processing systems often have their own network. Additionally, internal databases (ie financial databases) and other types of back office servers are often segmented from web servers and public facing systems. Access control lists in routers and firewalls control the traffic between various private networks. The ability to dynamically provision networks could be exposed to researchers as part of a private cloud but would require extensive access control within the middleware to prevent malicious users from interfering with the configuration of mission critical networks and services. Given the purpose of enterprise networks and services, the managers of such systems have strong incentives to not expose administrative interfaces to researchers and other end users to enable a “private cloud” utilizing the same administrative systems. In the future, it is possible that robust administrative systems, commonly referred to as middleware, will be developed that parallel the functionality of the most sophisticated cloud providers. When sophisticated middleware is available and research organizations adopt it, “private clouds” will be available for researchers within their own institutions or regional consortiums, assuming that it is cost effective.

Sophisticated cloud computing middleware is largely proprietary although open source platforms, such as OpenStack, do exist. However, since many organizations are already utilizing enterprise virtualization platforms, such as VMware, the task of enabling private clouds for research is more complicated than it might appear. Several questions that might be considered are:

- Should organizations set up a secondary private cloud platform for research workloads or utilize one platform for enterprise and research workloads?
- If one platform is selected, should this platform be a proprietary platform or an open source platform?
- Does the platform support an extensible interface to enable end users to configure network and virtual machines?
- How many physical servers should be devoted to each platform if two platforms are utilized?
- Will the private cloud platform and data centers be audited to any standards such as SAS 70 Type II or ISO 27001?

Most of these questions appear to be unresolved at many research organizations as the cloud platform software is still evolving and migrating existing systems is an expensive proposition. When platform software matures, private clouds may be constructed at research organizations, or research organizations may elect to utilize regional cloud providers or providers such as Amazon, depending on the cost and workload.

Commercial cloud providers offer an alternative to physical servers in local data centers by leveraging the organization’s architectural assistance to aid the design to ensure that it meets organizational policies. Advantageously, the systems are placed in an appropriate environment that is separate from the organization’s enterprise IT systems and research computing systems.

### Environment

Several major public cloud computing environments exist, such as Rackspace, Amazon’s EC2 service, and Verizon. These providers, and many smaller ones, allow customers to run an operating system of their choice and maintain full control of their operating system and network environment. These types of providers can broadly be classified as infrastructure as a service (IaaS) providers [8]. Platforms such as Microsoft Azure, often termed platform as a service (PaaS) [8], provide an application hosting environment, and the system administrators do not have direct control over the operating system or the network. Maintaining full control of the operating system allows system administrators of the leased compute environment to configure a host-based firewall, set up event logging, and perform any other customizations that are typical for their environment and threat model. This paper does *not* attempt to argue that it is impossible to achieve HIPAA compliance with platform as a service providers. We merely note that maintaining full control of an operating system and network allows the system administrators to set up servers in an environment that closely mirrors their internal network and data centers. For example, if a health organization typically uses software encryption to protect entire directories on a server, encryption of specific directories could also be set up in the operating systems hosted by IaaS providers, since the organization maintains full control.

While it may be possible to achieve HIPAA compliance when using PaaS providers, if the providers facilitate appropriate logging capability and server isolation, we believe that it is simpler to demonstrate HIPAA compliance with the traditional model of an organization controlling their own servers and network. Additionally, the legal and regulatory environment is more familiar with the concept of an organization maintaining full control of their operating system and network as opposed to new models where customers merely maintain control of the application layer. Information security guidance typically centers on a “defense in depth” strategy for protecting data, including the network, operating system, and application running on top of the operating system. Given the current regulatory environment, and the similarity between IaaS and organizations’ owned private data centers, we chose to use Amazon’s EC2 “Virtual Private Cloud” (VPC) as our platform. Amazon’s VPC has several important features that are noteworthy from a security perspective:

- Private subnets in Amazon’s environment with nonroutable Internet protocol (IP) addresses
- Option to assign public IP addresses to servers
- Ability to create a virtual private network (VPN) connection to Amazon to incorporate servers into the wide area network of an organization
- Combination of public IP addresses and VPN to create multitiered services
- Inbound and outbound stateful packet inspection firewall rules for each server group
- Ability to set up a host-based firewall (if supported in the operating system)
- Network access control lists (ACL) to control incoming and outgoing traffic at the subnet level
- Multiple network interfaces for each server to build sophisticated network architectures

These features, when combined, enable system architects to create flexible networks that can support a range of security requirements. If implemented correctly and accompanied by appropriate plans and monitoring, we think that these features allow a system to address the privacy and security rules mandated by HIPAA for electronic personal health information (e-PHI). Obviously, HIPAA is a complex set of laws, interpretations of the law, and technical implementations of the safeguards and consequently involves sophisticated risk analysis. The authors are not lawyers and advise the reader to seek appropriate legal counsel. The steps necessary to “ensure confidentiality, integrity, and availability of all e-PHI they create, receive, maintain or transmit”, part of the HIPAA Security Rule [9] for a computerized physician order entry (CPOE) system with 1000 users, is a significant undertaking. Ensuring the confidentiality, integrity, and availability of a research database for 5 users containing a limited subset of e-PHI data from a HIE is likely to be far simpler than designing and supporting an environment for a 1000-user CPOE. For example, 20 minutes of unscheduled downtime can likely be considered an acceptable risk for a research database. However, 20 minutes of unscheduled downtime for a CPOE could have disastrous results for a large medical center. The design of a scalable, available, and secure system for a CPOE in the cloud to support 1000 users is well beyond the scope of this paper. The intent of this paper is to analyze Amazon’s EC2 platform for use as a hosting environment for research datasets and applications [10], potentially containing e-PHI, referencing the US Department of Health and Human Services documents [7,9] where appropriate. Several other providers, such as Rackspace [11], also support private networking and security groups. The concept of VPC was proposed by researchers [12] before Amazon released their “Virtual Private Cloud” as a public product.

## Method

### Addressing the Security Rule

#### Risk Analysis and Management

Risk analysis and management affects the tradeoffs that must be made when selecting appropriate safeguards. First, the evaluation of the likelihood and impact of potential risk to e-PHI needs to be performed. For instance, outside parties may only obtain a copy of e-PHI data from the HIE, and the original e-PHI is not at risk for modification. Second, risk remediation safeguards need to be implemented. Third, a risk analysis and management plan should be documented and updated as the environment changes. Risk analysis and management should be familiar to information security departments, and a similar analysis process should be followed, regardless of the location of the virtual server (whether an infrastructure as a service provider such as Amazon is utilized or an organization’s private cloud).

#### Administrative Safeguards

The administrative safeguards follow from the “Risk Analysis and Management” section above. The organization with e-PHI must identify and analyze the risks to e-PHI and implement security measures to mitigate the risks. Additionally, the organization should designate a security official that is responsible for developing and implementing its security policies. An information access management policy should ensure that the Privacy Rule standards are met by enforcing the minimum disclosure necessary to support the task. Additionally, access to e-PHI data should be granted based on the user’s or recipient’s role. The security rule also requires that an organization must provide appropriate authorization and supervision of workforce members with access to e-PHI, train all members with access to e-PHI (according to the organization’s policies and procedures), and apply appropriate sanctions against members who violate the policies. HIPAA mandates that organizations periodically assess the effectiveness of their policies and procedures.

#### Physical Safeguards

Physical safeguards are composed of two broad items—“Facility Access and Control” and “Workstation and Device Security”. “Facility Access and Control” refers to “limiting physical access to its facilities while ensuring that authorized access is allowed.”

Infrastructure as a service offerings provide acceptable “Facility Access and Control” for the network (the network that supports the virtual servers) and virtual servers if the organization considers the providers’ standards acceptable. Amazon EC2’s service has completed a SAS 70 Type II audit, obtained ISO 27001 certification, and PCI level DSS validation as a level 1 service provider.

Our prototype’s environment is Amazon’s VPC, a configurable infrastructure as a service offering of Amazon’s EC2 service. The VPC enables customers to set up a private multitier network architecture, utilizing a subset of instance types available in EC2. The micro and the cluster compute instance types are currently unavailable in the VPC. This feature is not typically available to researchers at most research organizations unless they have a highly configurable cloud platform with extensive security and auditing tools.

Workstation and Device Security is also an important component of the security plan, and its importance cannot be overstated. If users’ workstations are in unsecured areas, without appropriate physical security, this presents a risk that has serious consequences. For example, if SSH keypairs are utilized without passphrases (or with weak passphrases) and stored on local file systems without any encryption, a basic attack could retrieve the private SSH key from a workstation when the user is not present, and the attacker could use the stolen credentials to impersonate the user. Similarly, keyboard logging software could be used to retrieve a user’s private key passphrase, or standard SSH password. Once a user’s credentials are impersonated, the attacker can gain access to e-PHI data on remote servers, potentially comprising e-PHI data. If user impersonation occurs, the ability to determine who inappropriately accessed e-PHI data is lost. One possible technical solution to reduce the risk of credential theft would be to implement smart cards.

There are many other types of attacks that can be launched from within an organization, and they are beyond the scope of this paper. The risk mitigation strategies are also beyond the scope of this paper. In addition, it would be impossible for the authors to comment on the readers’ environments because various organizations maintain different volumes of data, are subject to numerous types of laws (local, state, and obviously the federal HIPAA laws which mandate a minimum federal standard) and contracts, and experience their own threats. However, these examples serve to motivate the importance of workstation and device security.

Cloud computing does not remove the need to provide a trustworthy computing environment for workstations and other devices in use at an organization. It is paramount for access control, auditing, and integrity controls (below) to provide a trustworthy computing environment for user workstations so that user identity can be ascertained. User authentication is vital so that appropriate authorization decisions can be made by the servers containing e-PHI data. The organization’s information security and technology departments should provide a trustworthy environment for the organization’s devices and assist with the necessary risk and mitigation strategies that are appropriate for their specific environment.

The need for physical security also applies to the cloud administrator, privacy manager, security manager and any other key positions that have administrative control over systems with e-PHI data. Obviously, if the user credentials of the cloud manager are compromised, the trustworthiness of the audit records, e-PHI data, and the virtual servers is suspect. Our lab maintains a private data handling room for use by key personnel so that tampering with “important” workstations requires a higher level of determination and sophistication compared to a workstation in a public area shared by many individuals.

The workstation and device security section also includes requirements for the protection of e-PHI on electronic media. For example, when electronic media are transferred, removed, disposed, or re-used. Cloud-based block storage, such as Amazon’s Elastic Block Store (EBS) [13], should be treated the same as a magnetic or solid state disk. Before the volume is “disposed” by deleting it from an Amazon EC2 account, the volume should be sanitized using an approved wiping tool. This sanitization process can be achieved in the cloud by:

1. powering off the server S, but not terminating it
2. detaching the EBS volume, named J, from the original server instance S
3. attaching the EBS volume J as an additional volume to the wiping instance, W
4. mounting the volume, J, from within the operating system of the wiping instance, W
5. wiping the EBS volume, J, from within W using an appropriate tool

##### Next Steps for Workstation and Device Security

The organization should put in place a robust workstation and physical device security program (or re-evaluate their current program). This program should establish various classes of users and ensure an appropriate environment for each user class. For example, the user class that has administrative oversight of the cloud computing environment should be granted appropriate attention.

#### Technical Safeguards

**Figure 1:**
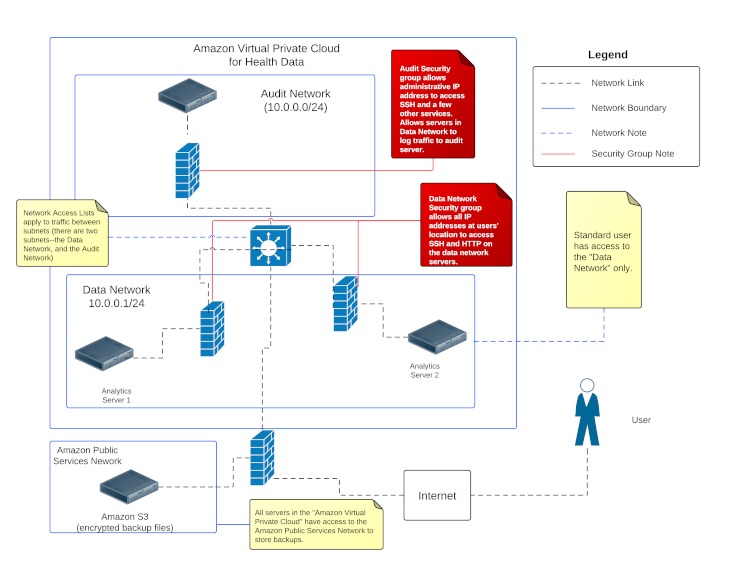
Our prototype environment for HIPAA data, utilizing two subnets, the “Data Network” for servers, and the “Audit Network” for an audit and monitoring server.

Technical safeguards are perhaps the most interesting of the Security Rule safeguards in an infrastructure as a service environment. Technical safeguards in a HIPAA environment consist of four primary categories: access control, audit controls, integrity controls, and transmission security. Access control ensures that only authorized persons are able to access e-PHI data. This is implemented in our environment with SSH2 RSA keypairs to link Unix login names to actual users, as SSH password login can be vulnerable to timing attacks [14]. Users created a keypair with a passphrase to protect the private key and were trained to store the private key on secure storage. Access control was strengthened by implementing host-based firewall rules (this also allows the capture of firewall audit records for audit and monitoring purposes), Amazon Security Group firewall rules (a stateful packet inspection firewall) that are applied to individual servers (see Tables 1 and 2), and network access control rules (nonstateful ACL rules) to control traffic between networks in the virtual private cloud (see Appendices 3-6). Our network configuration is presented in Figure 1. The audit server has its own private network and the corresponding security group is configured according to Table 1. The analytic servers are segmented to their own network, and the security group rules are presented in Table 2. For those interested in accessing SSH and other services over a VPN connection, an IPsec VPN can be constructed to the Amazon Virtual Private Cloud so that SSH and other traffic from an organization is tunneled over an encrypted connection. As a worst case scenario, assume that a vulnerability in an application level protocol is identified (such as SSH), and the password can be guessed based on the pattern of VPN traffic (which is itself based on the application level traffic, such as SSH) and passwords are obtained for this application. The malicious individual would not be able to use these credentials unless they could access the VPN connection to the VPC, bypass Amazon’s firewall and network access lists (assuming that all credentials obtained were used to access resources in the virtual private cloud and the passwords were not shared with any other systems external to the virtual private cloud), or collaborate with an internal user.

**Table 1:** Audit network security group rules.^a^

Item #	Direction	Source or Destination Address	Port/Protocol	Purpose
1	IN	Data Handling Room	22/TCP	SSH traffic for management
2	IN	Data Handling Room	8000/TCP	Splunk SSL connection for management of monitoring software
3	IN	10.0.1.0/24	10514/TCP	Syslog traffic from servers
4	OUT	0.0.0.0/0 (any network)	80/TCP	Retrieve operating system patches
5	OUT	10.0.0.2/32	53/UDP	DNS lookup
6	OUT	10.0.0.1/32	67/UDP	DHCP server in Amazon Virtual Private Cloud
7	OUT	0.0.0.0/0	123/UDP	NTP servers for time synchronization

^a^The Security Group is composed of two rule sets, inbound and outbound. The order of the rules is not important, but they are numbered for convenience in the table. When the direction is “IN”, the address field represents a source address. When the direction is “OUT”, the address field represents a destination address. Amazon’s web-based tools automatically populate the type of address field since each type of security group (IN or OUT) is stateful and automatically has an accompanying rule in the opposite direction to enable the traffic specified in that particular rule. An “OUT” rule in a stateful firewall can only control traffic to a destination.

**Table 2:** Security group for analytics servers.^a^

Item #	Direction	Source or Destination Address	Port/Protocol	Purpose
1	IN	129.74.0.0/16	22/TCP	SSH traffic for users at our campus
2	IN	129.74.0.0/16	443/TCP	SSL access for Apache web server to support web applications
3	OUT	HIE_IP/32	443/TCP	SSL access to retrieve data from HIE
4	OUT	10.0.0.10/32	10514/TCP	Syslog traffic to audit server
5	OUT	10.0.0.2/32	53/UDP	DNS lookup
6	OUT	10.0.0.1/32	67/UDP	DHCP server in Amazon Virtual Private Cloud
7	OUT	0.0.0.0/0	123/UDP	NTP servers for time synchronization

^a^The Security Group is composed of two rule sets, inbound and outbound. The order of the rules is not important, but they are numbered for convenience in the table. When the direction is “IN”, the address field represents a source address. When the direction is “OUT”, the address field represents a destination address. Amazon’s web-based tools automatically populate the type of address field since each type of security group (IN or OUT) is stateful and automatically has an accompanying rule in the opposite direction to enable the traffic specified in that particular rule. An “OUT” rule in a stateful firewall can only control traffic to a destination.

Amazon provides three methods of interacting with their management interface, which is utilized to create and terminate virtual servers and manage networking (including Security Group firewall rules and network access control lists). These methods are currently the Amazon AWS web-based management console, Amazon API Access Keys, and X.509 certificates. Since obtaining access to the API access key or X.509 private certificates would typically only require access to the cloud manager’s workstation (unless smartcards are utilized to store an X.509 certificate) , we elected to disable both of these access methods and exclusively utilize the AWS web-based client. The web-based client permits the use of multifactor authentication, which requires two pieces of information to authenticate the cloud manager—the cloud manager’s Amazon password (which only he/she knows) and the one-time password token from the cloud manager’s token device carried on a keychain. This approach is superior to one piece of information to prove identity and is likely warranted for the cloud manager and other personnel with high-level access to the configuration of the VPC. Impersonating the cloud manager would grant the attacker the ability to terminate the servers or at a minimum compromise their integrity by adjusting firewall rules, terminating the audit server, or perform other catastrophic acts. It is worth noting that it is possible to configure access lists to restrict API access to specific IP address ranges and times of day, complicating an attack using stolen API keys or X.509 certificates. The IP address restrictions could be used in combination with multifactor authentication to limit access for privileged accounts. However, this complicates failure modeling. For example, if a server is compromised and the cloud manager is on vacation and the company VPN is experiencing a failure, then the cloud manager would be unable to login to Amazon and power down a server or adjust the VPC configuration (assuming that the API calls are only accessible from within the organization).

##### Next Steps for Authentication and Authorization

Amazon provides many options for authentication to the EC2 management console, such as the Identity Access and Management services. These services allow organizations to utilize their own authentication systems to grant access to Amazon’s EC2 resources through identity federation for EC2 managers. The organization should determine the roles of the individuals that require access to the Amazon EC2 management console and the servers themselves. It is likely that organizations can readily utilize their own authentication and authorization systems for server and database login if they configure the private cloud at Amazon as an extension of their own network. Integrating internal user accounts with the EC2 management interface will likely require custom software development and may be unnecessary if only a limited number of users require access to the management console for VPC management.

Audit controls are processes and systems that collect access and activity information from systems that contain e-PHI. Our prototype implemented a central syslog server that was devoted to collecting and monitoring events from the project servers. SSH logins and firewall activity (from the host-based firewall) were logged to the syslog server. Our prototype system, containing deidentified health information, logged audit events according to Table 3. Splunk [15], a log management and monitoring tool, was installed on the logging server to monitor the audit event records. Databases that store e-PHI could potentially incorporate logging capability through stored procedures or utilize the database engine’s native logging capability (if it exists) and send these audit messages to the central logging server. For example, if e-PHI is accessed, deleted, or modified, an audit record could be generated and sent to the central logging server. The granularity of auditing should be selected with the consultation of the appropriate risk management and legal personnel.

**Table 3:** Audited events from servers.

Item #		Source	Event	Notes
1	SSH login	iptables host based firewall	SSH connection containing the source IP address	Traffic is logged before being accepted, ideally capturing any login attempts that cause the SSH daemon to fail.
2	SSH login	SSH daemon	SSH login, including type of authentication, and username	Provides more detail than 1, but occurs after the TCP connection is allowed.
3	Standard Redhat Linux System Events	Various Applications and System Services		
4	HTTPS request to HIE	Iptables host-based firewall		Establish baseline volume of requests and monitor for abnormal behavior

##### Next Steps for Auditing

Events should be defined according to the legal and business needs of the organization. Once these events are defined, the auditing and alerting software should be configured to alert the appropriate individuals when interesting events are detected.

#### Software Configuration

The server image was built with Amazon Linux AMI 2011.09 [16], and then we proceeded to install the Mirth Connect Server (using the command line installer), Apache, PHP, and MySQL, leaving these services turned off in chkconfig. The server image is simply a server without user accounts or other personalized information that can be readily cloned to create an arbitrary number of servers. After these tools were installed, we shut down the image machine and created an S3 snapshot of the machine so that we could launch instances based on this image (without a common MySQL password or SSL certificate). After instances were created and started from the image, we configured each instance with a unique MySQL password and Apache SSL certificate and created user accounts.

##### Next Steps for Software Configuration

The individual or team responsible for creating servers should obtain the necessary software and validate its authenticity. If multiple servers will be utilized for the same task the server can be cloned by creating an S3 image.

## Discussion

This paper presents a tutorial on how to duplicate our environment in Amazon’s EC2 “Virtual Private Cloud” to obtain data from a HIE. We believe this environment can be HIPAA compliant if best practices are followed as Amazon provides many features (not necessarily configured by default) that can be set up to suit a variety of security requirements. Information security references [17] have discussed HIPAA compliance for some time, and their methodologies can also be applied to new environments, such as the cloud.

Amazon’s documentation, to the best of our knowledge, does not clarify how the private networks are implemented. For instance, are they implemented using 802.1Q trunks, a proprietary overlay network, or something else entirely? The choice of an implementation presumably included the analysis of tradeoffs between performance, security, scalability, cost, and potentially other metrics of interest to Amazon. This tradeoff decision is of interest to system administrators so that they can determine if Amazon’s security model is an appropriate fit for their project.

Several improvements could be made to the VPC service related to the interaction of “Security Groups” and the services that are utilized by the Amazon Linux servers. One noticeable problem that we experienced was the dynamic nature of the update mirrors that are used to patch Amazon Linux. Ideally, there would be a small set of servers, with Amazon IP addresses, provided in a list, so that system administrators could easily implement outgoing traffic filtering in security groups and allow the update server traffic. Currently, since several mirrors are used, the process of identifying the IP addresses that could potentially be used for updates is haphazard and requires manual trial and error on the part of the system administrator, or alternatively the exemption of all traffic destined to port 80 during the update interval. A similar issue exists when attempting to use NTP servers, since public NTP server pools are typically outside of Amazon, and a given DNS hostname in the NTP pool can resolve to many addresses. Overall, the functionality of the “Security Groups” in the VPC is excellent, since it allows for inbound and outbound filtering, and its effectiveness would be enhanced if these issues are corrected.

Another issue that has more serious implications for auditing and contractual compliance, in our opinion, is the inability for the customer to save or otherwise access the events that are denied by the “Security Group” or “Network Access Control Lists”. This information could be valuable for information security professionals attempting to determine the volume and/or source of traffic that is targeted at their network. For example, port scans or other basic activities could be a sign of a pending attack or interest in the network by malicious users. While the host-based firewall rules could be used to signal suspicious behavior by internal users, the addition of audit records from the “Security Groups” or “Network ACLs” could corroborate the system level events in any legal action against internal users.

HIPAA does not mandate specific audit checklists for the setup of servers and networks and instead states that the security should be appropriate for the risk. HIPAA guidelines would be more useful to system administrators if additional guidance was provided regarding minimum standards. For example, what type of data should be audited to satisfy the requirement to “examine access and other activity in information systems that contain or use e-PHI” [9]? If these requirements were more clearly outlined, then it is likely that cloud providers would readily adopt the minimum requirements so that their customers could use their services for HIPAA compliance. The lack of clarity in HIPAA standards was noted by Wafa in a law review article [18].

There are several broad security considerations that should be mentioned when discussing a migration to cloud computing. First, cloud computing providers typically utilize virtualization to provide isolation [19]. If the underlying physical server is used to host computing resources for multiple customers, several types of side-channel attacks are possible [20]. Amazon offers an option to host virtualized compute servers on hardware that is dedicated (not shared) to a given customer’s resources for an additional cost. It is also important to note that side-channel vulnerabilities that exist because of the virtualization software (typically termed a hypervisor) will also likely exist in private data centers and private clusters, assuming that the “attacking” server (often shared on the same physical hardware as the target) is able to be accessed by an internal individual that wants something from the target server.

The reliability of the organization’s Internet connection is another important concern if the cloud based servers are primarily accessed from the organization. Based on our experience at our campus, our wide area network (and connection to local data centers) is more reliable than our connection to the commodity Internet. The organization should make the appropriate investments in redundant equipment and Internet connections if they decide to leverage cloud computing as a mission critical service. Amazon recently introduced [21] dedicated connections (1-10Gbps) to customers in specific geographic areas, which would decrease the reliance on commodity Internet connections, since the dedicated connection could be connected directly to the organization’s wide area network. It is likely that other providers will release similar products or at least attempt to address this issue.

The system architect must determine the risk and cost of the resulting event, should a side-channel attack occur in a large cloud, given the exposure of the system. For example, one might base the risk decision on whether the server is connected to the public Internet or accessible only through a VPN and the number and type of services that the server provides. For example, assuming that someone is able to demonstrate and carry out a side-channel attack to obtain RSA keys for server login, if the system is accessible only from a VPN, the other organization would not be able to easily utilize the stolen keys unless they could also gain access to the organization’s network and VPN.

### Conclusion

Ultimately, the system architect must attempt to answer the following questions. First, is their computing infrastructure safer at a cloud provider or in their own data center? Second, what is the budget that is allocated to a “more secure” environment, assuming the basic environment is HIPAA compliant? If the organization’s private data center frequently has power outages or only maintains simple backup tapes in the same facility as the servers, the system architect may decide that the benefits of cloud computing (demonstrable physical security, scalable and simple backups hosted in multiple locations, up to date patching of the hypervisor code) far outweigh the drawbacks (the remote possibility of sophisticated side-channel attacks). We suspect that the debate on these security issues is just starting and will continue.

In our experience, cloud computing has the capability to enable rapid provisioning of resources that can be configured to suit a variety of security requirements. Amazon’s Virtual Private Cloud offers one possibility to quickly provision sensitive data networks for research purposes. We believe that our prototype can be implemented in a HIPAA compliant manner (for research purposes) and that the discussion in this paper will provide a list of suggested improvements and considerations should readers wish to explore the implementation of a mission critical HIPAA workload in the cloud.

## Multimedia Appendix 1

Instructions for installing Mirth Connect on Redhat Enterprise Linux 5 or Redhat Enterprise Linux 6.

## Multimedia Appendix 2

Instructions for setting up Mirth Connect for an SSL connection to a HIE.

## Multimedia Appendix 3

Amazon Network Access Control List for incoming traffic to data network.

## Multimedia Appendix 4

Amazon Network Access Control List for outgoing traffic leaving data network.

## Multimedia Appendix 5

Amazon Network Access Control List for incoming traffic to the audit network.

## Multimedia Appendix 6

Amazon Network Access Control List for outgoing traffic leaving audit network.

## Abbreviations

ACL: access control list
CPOE: computerized physician order entry
DNS: domain name system
EBS: elastic block service
EHR: electronic health record
e-PHI: electronic personal health information
HIE: health information exchange
HIPAA: Health Insurance Portability and Accountability Act
IaaS: infrastructure as a service
NTP: network time protocol
PaaS: platform as a service
PHI: personal health information
SSH: secure shell
VLAN: virtual local area network
VPC: virtual private cloud
VPN: virtual private network

## References

[1] Amazon Web Services LLC (2011). Online.

[2] Mirth Corporation Mirth Connect, The Leading Open Source HL7 Interface Engine.

[3] iNTERFACEWARE Inc Components of an HL7 Message.

[4] Eveland J (2005). HL7 Segment : PID : Patient Information.

[5] US Department of Health & Human Services HIPAA Administrative Simplification Statute and Rules.

[6] US Centers for Medicare & Medicaid Services (2012). Transaction & Code Sets Standards.

[7] US Department of Health & Human Services Summary of the HIPAA Privacy Rule.

[8] Rimal BP, Eunmi C, Lumb I (2009). A Taxonomy and Survey of Cloud Computing Systems. Proceedings of the Fifth International Joint Conference on INC, IMS and IDC.

[9] US Department of Health & Human Services Summary of the HIPAA Security Rule.

[10] Ross SE, Johnson KB, Siek KA, Gordon JS, Khan DU, Haverhals LM (2011). Two complementary personal medication management applications developed on a common platform: case report. J Med Internet Res.

[11] Rackspace, US Inc. Managed Private Clouds.

[12] Wood T, Gerber A, Ramakrishnan KK, Shenoy P, Van der Merwe J (2009). The Case for Enterprise-Ready Virtual Private Clouds. Proceedings of the 2009 Conference on Hot Topics in Cloud Computing.

[13] Amazon Web Services LLC Amazon Elastic Block Store (EBS).

[14] Song DX, Wagner D, Tian X (2001). Timing analysis of keystrokes and timing attacks on SSH. Proceedings of the 10th USENIX Security Symposium.

[15] Splunk Inc Operational Intelligence, Log Management, Application Management, Enterprise Security and Compliance.

[16] Amazon Web Services LLC (2011). Amazon Linux AMI 2011-09 - Release Notes.

[17] MacLeod D, Geffert B, Deckter D, Tipton H, Krause M (2004). HIPAA 201: A Framework Approach to HIPAA Security Readiness. Information Security Management Handbook.

[18] Wafa T (2010). How the Lack of Prescriptive Technical Granularity in HIPAA Has Compromised Patient Privacy. North Illinois University Law Review.

[19] Li A, Yang X, Kandula S, Zhang M (2010). CloudCmp: comparing public cloud providers. Proceedings of the 10th ACM SIGCOMM Conference on Internet Measurement.

[20] Ristenpart T, Tromer E, Shacham H, Savage S (2009). Hey, you, get off of my cloud: exploring information leakage in third-party compute clouds. Proceedings of the 16th ACM Conference on Computer and Communications Security.

[21] Amazon Web Services LLC AWS Direct Connect.

