# Supplementary material for: Storing and Using Health Data in a Virtual Private Cloud
Source: J Med Internet Res. 2013 Mar 13;15(3):e63. doi: 10.2196/jmir.2076 (PMC3636251; doi:10.2196/jmir.2076)
Supplement: Supplementary file 1 [file jmir_v15i3e63_app1.pdf]

1. Install Java 1.6.x from Oracle or the operating system distribution's version of the openjdk. For Redhat Enterprise Linux 5, Java 1.6.0 can be installed with *yum install java-1.6.0-openjdk*.

2. Download the Mirth Connect installer for Unix (at the time of writing, the download link is *http://downloads.mirthcorp.com/connect/2.2.0.5828.b1215/mirthconnect-2.2.0.5828.b1215-unix.sh*).

3. Start the installer with *./mirthconnect-2.2.0.5828.b1215-unix.sh* with sudo or from the root account and click *Next*. Accept the license agreement on the next page and click *Next*.

4. Select the destination directory, such as */opt/mirthconnect*. Click *Next*.

5. Click *Next* on the following screen after leaving all of the components selected for installation.

6. Click *Next* to accept the destination directory for symbolic links.

7. Review the default listing of ports and click *Next*.

8. Review the security settings, adjusting as necessary to meet organizational password requirements and legal requirements. Click *Next*.

9. Review and adjust the directory locations for "Application Data" and "Logs".

10. Click *Next* after leaving the "Install Service" item selected.

11. Deselect the "Yes, I would like to view the readme" box and click *Next*.
