# Supplementary material for: Storing and Using Health Data in a Virtual Private Cloud
Source: J Med Internet Res. 2013 Mar 13;15(3):e63. doi: 10.2196/jmir.2076 (PMC3636251; doi:10.2196/jmir.2076)
Supplement: Supplementary file 2 [file jmir_v15i3e63_app2.pdf]

1. Install MySQL Server. In Redhat Enterprise Linux 5 or Redhat Enterprise Linux 6, this is done with "*sudo yum install mysql-server mysql-client*". In Ubuntu, "*sudo apt-get install mysql-server mysql-client*" will install MySQL server.

2. Login to MySQL as the root database user, and setup a database for Mirth. Without MySQL configuration, the database root password is non-existent (a strong password should be set for production use). Replace "X" below with a password of your choice. "*mysql -u root –p mysql_root_password*"
   "*CREATE DATABASE mirthdb DEFAULT CHARACTER SET utf8;*"
   "GRANT ALL ON mirthdb.* TO mirthuser@'localhost' IDENTIFIED BY 'X' WITH GRANT OPTION;"
   "GRANT ALL ON mirthdb.* TO mirthuser@'%' IDENTIFIED BY 'X' WITH GRANT OPTION;"

3. Stop the Mirth Connect Server if it is running by visiting the *Mirth Connect Server Manager* and stopping the service (alternatively, with command line access only, the process *mcserver* can be safely killed).

4. Modify the Mirth configuration file (conf/mirth.properties file in the Mirth directory) to contain the following in the database area of the file:
   database = mysql
   database.url = jdbc:mysql://localhost:3306/mirthdb
   database.username =mirthuser
   database.password =X

5. Comment out (by prepending the line with *#*) the line containing derby database information.

6. Alternatively (in place of steps 4 and 5), if an X11 session is available, place the MySQL username you created in the installation instructions (*mirthuser* in our example) and the MySQL password in the *Database* tab of the *Mirth Connect Server Manager.*

7. Start the Mirth Connect Server by clicking *Start* on the *Service* tab. The "mirthdb" database should now have tables (this can be verified by logging in as mirthuser echo *"show tables" | mysql –u mirthuser –pX*. This will return a list of tables (if they have been created).

8. The administrative console can be accessed at http://localhost:8080.

9. The default username is *admin* and the default password is *admin*. This is personalized at the first login to the administrative console.

10. Install the SSL plugin for Mirth (this requires a license) by visiting the "Extensions" tab and selecting "Install Extension from File System", select the SSL plugin's filename (similar to *ssl-2.1.1.b774.zzz.zip*) and click *Install*.

11. Export the SSL certificate from your HIE (or obtain it from them).

12. Generate a Keystore with portecle (portecle.sourceforge.net) and create an RSA public/private key pair with key size of 2048 bits.

13. On the *Settings* tab in Mirth Connect Administrator, visit the SSL tab, and enter the path to the keystore and the passwords. Save the settings changes.

14. Go to *channels* and *Add a channel*. Enter a name for the channel, such as *My_HIE* Under the destinations tab use an HTTP sender as a *Connection Type*. For URL, enter the URL of your HIE's server, following the format *https://server:port*. In content put "${payload}". Now go to E*dit Transformers*. Click *Add new step*. Change the type to JavaScript by clicking in the area under *Type*. Put *channelMap.put('payload',"\x0B"+messageObject.getRawData()+"\x1C\x0D");* in the text field. Click *Back to Channel* and Click *Save Changes*.

15. Set up another channel by visiting *Channels* and clicking *New Channel*. Name the channel (for example, *HIE_DATA*), perhaps to denote that it is data being brought into your system. Under *Source* select *Channel Reader*. Under *Destinations* select *File Writer*. Enter a directory path and file name. Drag *Raw Data* from the box at the right to the template section. Save your changes by clicking *Save Changes*.

16. Go to *Settings*, then *SSL Manager*. Check the *Enable SSL* box for the channel with the http sender. Select the keystore you previously created for this HIE.

17. Go back to the http sender channel and under the *Destinations* tab set the *Send Response to:* to the other channel (the name that you utilized should be available as an option).

18. In the channels area choose *Redeploy All*. Restart the Mirth Server by going to Mirth Manager (if you have an X11 session to the server) and restart the server. Alternatively, you can manually kill the process and restart the server with */opt/mirthserver/mcserver.*

19. Go to *Dashboard*. Double click on the channel that has the HTTP sender. Click send message. Send a test message according to instructions provided by your HIE. This will likely be an HL7 message, and should conform to the specifications. Our test involved sending a request for a fake patient's information to the HIE, with the message response formatted according to an HL7 message segment for patient identification [4]. General information regarding HL7 messages is available from internet resources [3].
