# Supplementary material for: Storing and Using Health Data in a Virtual Private Cloud
Source: J Med Internet Res. 2013 Mar 13;15(3):e63. doi: 10.2196/jmir.2076 (PMC3636251; doi:10.2196/jmir.2076)
Supplement: Supplementary file 3 [file jmir_v15i3e63_app3.pdf]

Rule #	Port (Service)	Protocol	Source	Allow/Deny	Notes
100	1024 - 65535	TCP	10.0.0.10/32	ALLOW	Matching rule to allow return syslog traffic
102	53 (DNS)	UDP	10.0.0.2/32	ALLOW	Matching rule to allow return traffic from DNS server
108	ALL	ALL	10.0.0.0/16	DENY	Deny all traffic from other subnets
110	22 (SSH)	TCP	129.74.0.0/16	ALLOW	Allow incoming SSH from campus
111	1024 - 65535	TCP	0.0.0.0/0	ALLOW	Matching rule to allow HTTP return traffic
112	22 (SSH)	TCP	66.254.224.0/19	ALLOW	Allow incoming SSH from campus
113	22 (SSH)	TCP	66.205.160.0/20	ALLOW	Allow incoming SSH from campus
120	123	UDP	0.0.0.0/0	ALLOW	Allow outgoing NTP to public NTP servers
130	1024 - 65535	TCP	HIE_IP/HIE_IP_MASK	ALLOW	Matching rule to allow HTTPS to HIE
140	443 (HTTPS)	TCP	129.74.0.0/16	ALLOW	Allow incoming HTTPS from campus
141	443 (HTTPS)	TCP	66.254.224.0/19	ALLOW	Allow incoming HTTPS from campus
142	443 (HTTPS)	TCP	66.205.160.0/20	ALLOW	Allow incoming HTTPS from campus
*	ALL	ALL	0.0.0.0/0	DENY	Automatic deny rule
