# Supplementary material for: Storing and Using Health Data in a Virtual Private Cloud
Source: J Med Internet Res. 2013 Mar 13;15(3):e63. doi: 10.2196/jmir.2076 (PMC3636251; doi:10.2196/jmir.2076)
Supplement: Supplementary file 4 [file jmir_v15i3e63_app4.pdf]

Rule #	Port (Service)	Protocol	Destination	Allow/Deny	Notes
100	10514	TCP	10.0.0.10/32	ALLOW	syslog traffic to syslog server
102	53 (DNS)	UDP	10.0.0.2/32	ALLOW	DNS lookup traffic to VPC's DNS server
103	ALL	ALL	10.0.2.0/24	DENY	deny traffic from other subnet in the VPC
108	ALL	ALL	10.0.0.0/16	DENY	deny traffic from other subnets in the VPC
110	1024 - 65535	TCP	129.74.0.0/16	ALLOW	Matching rule to allow outgoing SSH to our campus
111	80 (HTTP)	TCP	0.0.0.0/0	ALLOW	Allow HTTP for server patching
112	1024 - 65535	TCP	66.254.224.0/19	ALLOW	Matching rule to allow outgoing SSH return traffic to our campus
113	1024 - 65535	TCP	66.205.160.0/20	ALLOW	Matching rule to allow outgoing SSH return traffic to our campus
120	123	UDP	0.0.0.0/0	ALLOW	Allow NTP (time server) synchronization traffic
130	443 (HTTPS)	TCP	HIE_IP/HIE_SUBNET	ALLOW	Allow HTTPS connection to HIE IP
140	1024 - 65535	TCP	129.74.0.0/16	ALLOW	Matching rule to allow outgoing HTTPS return traffic to our campus
141	1024 - 65535	TCP	66.254.224.0/19	ALLOW	Matching rule to allow outgoing HTTPS return traffic to our campus
142	1024 - 65535	TCP	66.205.160.0/20	ALLOW	Matching rule to allow outgoing HTTPS return traffic to our campus
*	ALL	ALL	0.0.0.0/0	DENY	Automatic last rule.
