# Supplementary material for: Storing and Using Health Data in a Virtual Private Cloud
Source: J Med Internet Res. 2013 Mar 13;15(3):e63. doi: 10.2196/jmir.2076 (PMC3636251; doi:10.2196/jmir.2076)
Supplement: Supplementary file 5 [file jmir_v15i3e63_app5.pdf]

Rule #	Port (Service)	Protocol	Source	Allow/Deny	Notes
100	10514	TCP	10.0.0.0/16	ALLOW	Incoming syslog traffic from all subnets in the VPC
108	ALL	ALL	10.0.0.0/16	DENY	Deny traffic from other subnets in the VPC
110	22 (SSH)	TCP	DATA_HANDLING_IP/32	ALLOW	Permit SSH from data handling room
111	1024 - 65535	TCP	0.0.0.0/0	ALLOW	Matching rule to permit incoming HTTP traffic
112	8000	TCP	DATA_HANDLING_IP/32	ALLOW	Permit incoming HTTP traffic for Splunk monitoring
120	123	UDP	0.0.0.0/0	ALLOW	Matching rule to permit incoming NTP traffic
*	ALL	ALL	0.0.0.0/0	DENY	Automatic deny rule
