# Supplementary material for: Storing and Using Health Data in a Virtual Private Cloud
Source: J Med Internet Res. 2013 Mar 13;15(3):e63. doi: 10.2196/jmir.2076 (PMC3636251; doi:10.2196/jmir.2076)
Supplement: Supplementary file 6 [file jmir_v15i3e63_app6.pdf]

Rule #	Port (Service)	Protocol	Destination	Allow/Deny	Notes
100	1024 - 65535	TCP	10.0.0.0/16	ALLOW	Matching rule to permit syslog return traffic
102	ALL	ALL	10.0.1.0/24	DENY	Deny outgoing traffic to data network subnet in VPC
103	ALL	ALL	10.0.2.0/24	DENY	Deny outgoing traffic to future network subnet in VPC
108	ALL	ALL	10.0.0.0/16	DENY	Deny outgoing traffic to other subnets in VPC
110	1024 - 65535	TCP	DATA_HANDLING_IP/32	ALLOW	Matching rule to permit SSH return traffic to data handling room
111	80 (HTTP)	TCP	0.0.0.0/0	ALLOW	Permit HTTP for operating system patching
112	1024 - 65535	TCP	DATA_HANDLING_IP/32	ALLOW	Matching rule to permit outgoing Splunk monitoring traffic (HTTP based)
120	123	UDP	0.0.0.0/0	ALLOW	Permit NTP traffic
*	ALL	ALL	0.0.0.0/0	DENY	Automatic deny rule
